# Development of a Risk Prediction Model for Adverse Skin Events Associated with TNF-α Inhibitors in Rheumatoid Arthritis Patients

**DOI:** 10.3390/jcm13144050

**Published:** 2024-07-11

**Authors:** Woorim Kim, Soo-Jin Oh, Hyun-Jeong Kim, Jun-Hyeob Kim, Jin-Yeon Gil, Young-Sook Ku, Joo-Hee Kim, Hyoun-Ah Kim, Ju-Yang Jung, In-Ah Choi, Ji-Hyoun Kim, Jinhyun Kim, Ji-Min Han, Kyung-Eun Lee

**Affiliations:** 1 College of Pharmacy, Kangwon National University, Chuncheon 24341, Republic of Korea; 2 College of Pharmacy, Chungbuk National University, Cheongju 28160, Republic of Koreajmhan@chungbuk.ac.kr (J.-M.H.); 3 Department of Pharmacy, Chungbuk National University Hospital, Cheongju 28644, Republic of Korea; 4 College of Pharmacy, Ajou University, Suwon 16499, Republic of Korea; 5 Department of Rheumatology, Ajou University School of Medicine, Suwon 16499, Republic of Korea; 6 Division of Rheumatology, Department of Internal Medicine, Chungbuk National University Hospital, Cheongju 28644, Republic of Korea; 7 Department of Internal Medicine, College of Medicine, Chungbuk National University, Cheongju 28644, Republic of Korea; 8 Department of Internal Medicine, Chungnam National University College of Medicine, Daejeon 35015, Republic of Korea

**Keywords:** genome-wide association study, TNF-α inhibitor, skin and subcutaneous tissue-related complications, risk prediction

## Abstract

**Background:** Rheumatoid arthritis (RA) is a chronic inflammatory disorder primarily targeting joints, significantly impacting patients’ quality of life. The introduction of tumor necrosis factor-alpha (TNF-α) inhibitors has markedly improved RA management by reducing inflammation. However, these medications are associated with adverse skin reactions, which can vary greatly among patients due to genetic differences. **Objectives:** This study aimed to identify risk factors associated with skin adverse events by TNF-α in RA patients. **Methods:** A cohort study was conducted, encompassing patients with RA who were prescribed TNF-α inhibitors. This study utilized machine learning algorithms to analyze genetic data and identify markers associated with skin-related adverse events. Various machine learning algorithms were employed to predict skin and subcutaneous tissue-related outcomes, leading to the development of a risk-scoring system. Multivariable logistic regression analysis identified independent risk factors for skin and subcutaneous tissue-related complications. **Results:** After adjusting for covariates, individuals with the TT genotype of rs12551103, A allele carriers of rs13265933, and C allele carriers of rs73210737 exhibited approximately 20-, 14-, and 10-fold higher incidences of skin adverse events, respectively, compared to those with the C allele, GG genotype, and TT genotype. The machine learning algorithms used for risk prediction showed excellent performance. The risk of skin adverse events among patients receiving TNF-α inhibitors varied based on the risk score: 0 points, 0.6%; 2 points, 3.6%; 3 points, 8.5%; 4 points, 18.9%; 5 points, 36.7%; 6 points, 59.2%; 8 points, 90.0%; 9 points, 95.7%; and 10 points, 98.2%. **Conclusions:** These findings, emerging from this preliminary study, lay the groundwork for personalized intervention strategies to prevent TNF-α inhibitor-associated skin adverse events. This approach has the potential to improve patient outcomes by minimizing the risk of adverse effects while optimizing therapeutic efficacy.

## 1. Introduction

Rheumatoid arthritis (RA) is a chronic inflammatory condition primarily affecting the hands, wrists, and feet, potentially leading to joint damage and functional limitations. Those with RA often experience reduced quality of life across physical health, autonomy, environment, and personal beliefs [1]. Typically, RA patients have a shorter life expectancy than the general population [2]. Therefore, initiating and maintaining effective treatment to achieve sustained remission is critical.

The introduction of tumor necrosis factor-alpha (TNF-α) inhibitors has revolutionized RA treatment, paving the way for new biologic disease-modifying antirheumatic drugs (DMARDs) [3]. TNF-α, a pro-inflammatory cytokine, plays a pivotal role in RA. Blocking TNF-α in RA patients significantly reduces concentrations of interleukin (IL)-1, IL-6, IL-8, and granulocyte–macrophage colony-stimulating factor in synovial cell cultures compared to the placebo group [4]. Consequently, TNF-α inhibition proves more effective in controlling inflammation than targeting other highly concentrated synovial fluid cytokines, including IL-1 [5].

TNF-α therapy, however, can significantly impact the immune system and trigger autoimmune-related adverse events, particularly skin and subcutaneous tissue disorders. Reported cutaneous side effects from TNF-α blockers include injection site reactions, infusion reaction skin manifestations, infections, non-melanoma skin cancer, and psoriasis [6]. Studies in rheumatology indicated nearly a quarter of patients on anti-TNF-α therapy experience cutaneous complications [7,8], and a meta-analysis revealed a high incidence of such events in these medication recipients [9].

The rise in cutaneous adverse events linked to anti-TNF therapy might be influenced by factors such as other immunosuppressive drugs, aging, or concurrent conditions like chronic lung diseases and diabetes [10]. Severe cutaneous side effects have been reported in approximately 21–34% of patients undergoing TNF-α inhibitor treatment of rheumatologic illnesses or inflammatory bowel diseases, leading to treatment discontinuation in some instances [11,12,13,14]. Despite these observations, the impact of genetic factors on cutaneous complications related to TNF-α inhibitors remains inadequately explored. To bridge this gap, machine learning techniques were employed to investigate genetic and clinical aspects associated with skin adverse events, ultimately developing a risk score.

## 2. Methods

Patients who had been prescribed TNF-α inhibitors (adalimumab, etanercept, golimumab, or infliximab) were enrolled between July 2017 and August 2021 at Ajou University Hospital, Chungbuk National University Hospital, and Chungnam National University Hospital. In total, 117 patients were enrolled initially, and after excluding 4 patients with incomplete medical data, 113 patients were included in the analysis. Data on sex, age, weight, height, DAS28 and its subcomponents, duration of RA, rheumatoid factor, anti-cyclic citrullinated peptide antibodies, concomitant medications, and comorbidities were collected from electronic medical records. Skin and subcutaneous tissue-related adverse events were classified as grade 2 or higher according to the Common Terminology Criteria for Adverse Events (CTCAE), version 5.0 [15], which included alopecia, body odor, bullous dermatitis, dry skin, eczema, erythema multiforme, erythroderma, fat atrophy, hair color changes, hair texture abnormal, hirsutism, hyperhidrosis, hyperkeratosis, hypertrichosis, hypohidrosis, lipohypertrophy, nail changes, nail discoloration, nail loss, nail ridging, skin pain, palmar–plantar erythrodysesthesia syndrome, photosensitivity, pruritus, purpura, acneiform rash, maculo-papular rash, scalp pain, skin atrophy, skin hyperpigmentation, skin hypopigmentation, skin induration, skin ulceration, Steven–Johnson syndrome, subcutaneous emphysema, telangiectasia, toxic epidermal necrolysis, and urticaria [15].

This study was approved by the ethics committees of Ajou University Hospital (AJIRB-BMR-OBS-17-153), Chungbuk National University Hospital (2017-06-011-004), and Chungnam National University Hospital (2019-06-029-007), and patients provided written informed consent for participation. The study was conducted according to the principles of the Declaration of Helsinki (2013).

### 2.1. Genotyping Methods

Genome-wide genotyping was performed using an Affymetrix Axiom Korean Chip (v1.1) on more than 750,000 markers. A QIAamp DNA Blood Mini Kit (Qiagen GmbH, Hilden, Germany) was used per the manufacturer’s protocol to isolate genomic DNA from ethylenediaminetetraacetic acid–blood samples. The selection of variants was based on a genotype call rate of over 95% in a quality control test. SNPs with Hardy–Weinberg equilibrium at a threshold of *p* > 10^−4^ and high minor allele frequency (>10%) were tested in our GWAS; 262,242 SNPs remained with a Bonferroni threshold of 1.90 × 10^−7^. We selected SNPs with a *p*-value threshold of 10^−5^ to include signals approaching genome-wide significance, indicating suggestive relevance to the association.

### 2.2. Statistical Analysis and Machine Learning Methods

The Chi^2^ test or Fisher’s exact test was used to compare categorical variables between patients with and without skin and subcutaneous tissue-related adverse events. Independent risk factors for these adverse events were examined through multivariable logistic regression analysis using backward elimination. The multivariable analysis included factors with *p*-values below 0.05 in the univariate analysis and clinically relevant confounders such as age and sex. We assessed the model’s goodness of fit using the Hosmer–Lemeshow test. Statistical analyses were conducted using IBM SPSS Statistics, version 20 software (International Business Machines Corp., New York, NY, USA).

The following machine learning methods were used to predict skin and subcutaneous tissue-related outcomes: multivariate logistic regression, elastic net, random forest, and support vector machine (SVM). We implemented all methods with the caret R package. The area under the receiver operating characteristic (AUROC) and area under the precision–recall curve (AUPRC) were used to assess whether a risk factor can predict complications, along with its 95% confidence interval (CI) for each machine learning prediction model. Machine learning analyses were performed using R software version 3.6.0 (R Foundation for Statistical Computing, Vienna, Austria). Internal validation was conducted to measure the performance of each model. The dataset (*n* = 113) was randomly divided into sub-cohorts to develop a model and evaluate the prediction process. The generalizability of the model was estimated using the 5-fold cross-validation method. Each cross-validation iteration was repeated 100 times to assess the power of the machine learning models.

### 2.3. Development of a Risk-Scoring System

A new risk-scoring model was developed, incorporating risk factors from the derivation cohort. Variables less strongly linked were removed stepwise from the model when the *p* value was less than 0.10. All variables included in the final statistical model were incorporated into a scoring model, accepting that some variables were not statistically significant but clinically significant. The adjusted odds ratios from multivariable logistic regression analyses were divided by the smallest odds ratio among all variables to calculate a risk score. Afterward, quotients were rounded to the nearest integer to develop a scoring system to predict skin and subcutaneous tissue-related complications. To validate the risk-scoring system, we assessed its performance by calculating the AUROC. The expected risk of cutaneous complications was calculated by logistic regression analysis.

## 3. Results

One hundred seventeen patients were enrolled in the cohort during the study period. After excluding 4 patients with incomplete medical data, 113 patients were included in the analysis. The distribution of specific TNF-α inhibitors used was as follows: 45 patients with adalimumab, 12 patients with baricitinib, 29 patients with etanercept, 31 patients with golimumab, and 14 patients with infliximab. The duration of TNF-α inhibitor usage varied among patients and is continuously ongoing. Patients were monitored every 6 months, with treatment durations ranging from 1 month to 134 months, and an average treatment duration of approximately 35 months. The mean age of the patients included in the study was 52.3 years, ranging from 20 to 82 years, with 91 individuals (80.5%) being female. Eleven patients (9.7%) experienced skin and subcutaneous tissue-related adverse events after receiving anti-TNF-α treatment. The majority of the baseline characteristics of patients did not show statistical significance according to adverse skin outcomes (Table 1). Patients receiving hydroxychloroquine revealed approximately 4.6 times increased skin-related adverse events than those without the medication (*p* = 0.042). Patients’ disease severity did not differ significantly based on their baseline disease activity score (DAS)-28 (Supplementary Table S1).

**Figure 1:**
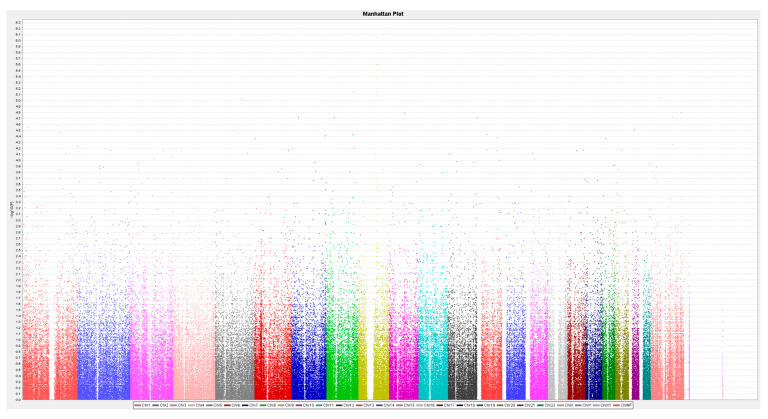
Manhattan plot.

By employing the feature selection methods in the Chi^2^ allelic test conducted in PLINK, we identified 12 SNPs with a *p*-value less than 10^−5^ for the adverse event phenotypes (Figure 1). Statistically significant associations were observed between genotypes and adverse skin outcomes for the following SNPs: rs12551710, rs116872662, rs74911558, rs4504713, rs17086428, rs13265933, rs12551103, rs2889244, rs75858517, rs79503383, rs73210737, and rs920388. Eventually, four SNPs—rs12551103 in FERM Domain Containing 3 (FRMD3), rs13265933 in RP11-1082L8.3, rs73210737 in RP11-245M24.1, and rs920388 at 33 kb 3′ of RP11-425E13.1—were selected for further analysis after excluding SNPs that were in high linkage disequilibrium (LD) with each other (r^2^ > 0.8).

For rs12551103, 4 of 6 patients (66.7%) with the TT genotype showed skin and subcutaneous tissue-related adverse events, whereas 7 of 107 patients (6.54%) with the C allele experienced such events (OR 28.57; 95% CI 4.437–183.964). For rs13265933, patients with the variant allele (A allele) showed more skin and subcutaneous tissue-related outcomes than wild-type homozygotes (the GG genotype) (28.1% vs. 2.5%; OR 15.46; 95% CI 3.117–76.633). For rs73210737, 4 of 93 patients (4.3%) with the TT genotype had adverse events, whereas 7 out of 20 patients (35.0%) with the C allele had them (OR 11.98; 95% CI 3.077–46.649). For rs920388, patients with the TT genotype showed higher skin and subcutaneous tissue-related adverse events than those with the G allele (75.0% vs. 7.3%; OR 37.86; 95% CI 3.523–407.155) (Table 2 and Table 3).

Multivariable logistic regression included age, sex, and factors with *p* < 0.05 in univariate analysis (Table 3). Of the four SNPs included in the analysis, rs12551103, rs13265933, and rs73210737 were significantly associated with skin and subcutaneous tissue-related complications (*p* < 0.05). After adjusting for related covariates, the TT genotype carriers of rs12551103 exhibited an approximately 20-fold higher incidence of cutaneous complications than C allele carriers. For rs13265933, A allele carriers showed about a 14-fold higher incidence of cutaneous outcomes than the GG genotype carriers. The C allele carriers of rs73210737 had a 10-fold higher incidence of cutaneous complications than those with the TT genotype. The Hosmer–Lemeshow test showed that the fitness of the multivariable analysis model was satisfactory (χ^2^ = 1.754, 2 degrees of freedom, *p* = 0.416).

**Figure 2:**
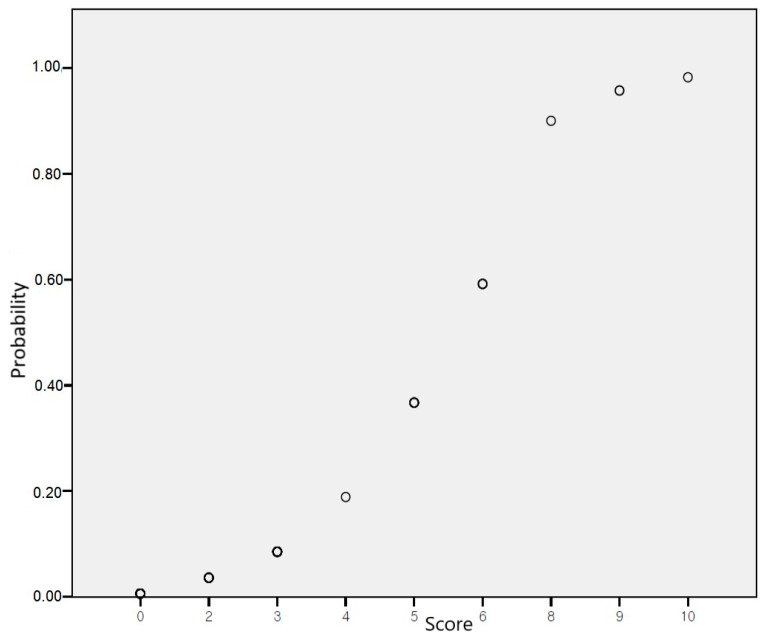
Risk probability according to scores using logistic regression.

For risk score prediction through machine learning algorithms, variables from the final step of multivariable logistic regression analysis with backward elimination were included as features. The adjusted odds ratios of all variables in the model were modified into an integer risk score (Table 3). The TT genotype carriers of rs12551103 (4 points), A allele carriers of rs13265933 (3 points), C allele carriers of rs73210737 (2 points), and TT genotype carriers of rs920388 (3 points) were integrated into the risk-scoring system. The risk scores ranged from 0 to 12; patients receiving TNF-α inhibitors with 0, 2, 3, 4, 5, 6, 8, 9, and 10 points showed about 0.6%, 3.6%, 8.5%, 18.9%, 36.7%, 59.2%, 90.0%, 95.7%, and 98.2% risk of skin and subcutaneous tissue-related adverse events, respectively. A logistic regression curve obtained by mapping the scores to risk scores is presented in Figure 2, and the risk probability according to scores using logistic regression is shown in Supplementary Table S2. To evaluate the predictive accuracy of the risk-scoring model for TNF-α inhibitor-induced skin and subcutaneous tissue-related adverse events, an AUROC of 0.91 (95% CI 0.796–1.000) was calculated.

The average AUROC and AUPRC values across 100 random iterations were calculated after 5-fold cross-validation of multivariate logistic regression, elastic net, random forest, and SVM models (Supplementary Table S3). The AUROC values with approximately 0.90 for machine learning algorithms indicated good performances of the model (95% CI 0.630–0.976, 0.679–0.992, 0.633–0.970, 0.620–0.970, and 0.636–0.969, respectively). The AUPRC values of multivariate logistic regression, elastic net, random forest, linear kernel SVM, and radial kernel SVM were 0.80, 0.84, 0.80, 0.79, and 0.80, respectively (95% CI 0.630–0.976, 0.679–0.992, 0.633–0.970, 0.620–0.970, and 0.636–0.969, respectively). Details of machine learning specifics are included in Supplementary Table S4.

## 4. Discussion

The main finding of this study was that rs12551103 in FRMD3, rs13265933 in RP11-1082L8.3, and rs73210737 in RP11-245M24.1 were factors associated with adverse skin outcomes of TNF-α inhibitor. A risk score model incorporating risk factors (rs12551103, rs13265933, rs73210737, and rs920388) was developed and had a reasonable prediction rate with an AUROC value of 0.91. The logistic regression analysis revealed that the risk of skin adverse events increased as patients receiving TNF-α inhibitors scored higher on a scale of 0 to 10, with corresponding risks ranging from 0.6% to 98.2%.

TNF-α inhibitors are linked to dermatological complications [16]. In one study, dermatological adverse events occurred in almost one-third of patients receiving anti-TNF-α therapy [17]. Another study showed that one in five patients treated with TNF-α inhibitors suffered dermatological complications after a 14-year follow-up [11]. Even though the exact mechanisms are still unknown, evidence suggests that a decrease in TNF-α activates autoreactive T cells and increases interferon activity, as well as the levels of pro-inflammatory cytokines, such as IL-12, -17, and -23 [12,18,19,20]. The discontinuation rate of TNF-α inhibitors due to skin-related adverse events varies across different studies, ranging from 15% to 40% [21]. In addition, numerous case reports have provided evidence suggesting a potential association between the utilization of TNF-α inhibitors and a higher occurrence of non-melanoma skin cancer (NMSC), specifically squamous cell carcinoma [7,22]. A recent meta-analysis of randomized controlled trials involving TNF-α inhibitors revealed a relative risk of 2.02 for NMSCs [23].

Our study discovered novel genetic markers associated with skin adverse outcomes of TNF-α inhibitors. rs12551103 is an intronic SNP in FRMD3. An expression quantitative trait locus (eQTL) analysis was performed using the Genotype-tissue Expression (GTEx) portal to investigate variations in gene expression according to genotypes. The expression of FRMD3 in fibroblasts was higher in TT genotype carriers of rs12551103 than in the C allele carriers (normalized effect size (NES) = 0.26, *p* = 9.6 × 10^−7^). Since FRMD3 is expressed in dermal fibroblasts [24], its overexpression may alter dermal function, thereby increasing the incidence of skin and subcutaneous tissue-related complications in patients receiving TNF-α inhibitors. rs13265933 is an intronic SNP in the uncharacterized locus LOC105375743 that is rarely studied. eQTL results showed that A allele carriers of rs13265933 had higher gene expression in fibroblasts than GG genotype carriers (NES = 0.21, *p* = 6.0 × 10^−11^). Similar to the case of rs12551103, overexpression of this gene may affect dermal function, resulting in more skin and subcutaneous tissue complications. FRMD3 plays an essential role in dermatologic adverse events. A whole-genome sequencing study revealed that FRMD3 is associated with eczema herpeticum [25]. Another study showed that FRMD3 is related to the risk of keloid formation [26]. In this study, we identified novel SNPs, rs73210737 and rs920388, associated with skin and subcutaneous tissue adverse events in RA patients receiving anti-TNF-α treatment. Further investigations should determine the mechanisms of such complications and genetic variations.

Machine learning methods have been on the rise for making decisions and clinical predictions. Compared to traditional predictive models that use particular variables for calculation, machine learning approaches help develop novel risk prediction models. However, no machine learning method has been developed for RA patients treated with TNF-α inhibitors to predict cutaneous toxicities. This study showed that each of the tested machine learning algorithms trained for risk prediction had good performance: the AUROC values were 0.90 (95% CI 0.630–0.976) for multivariate logistic regression, 0.90 (0.679–0.992) for the elastic net, 0.88 (0.636–0.969) for radial-kernel SVM, 0.89 for (0.633–0.970) for random forest, and 0.89 (0.620–0.970) for linear-kernel SVM. Machine learning methods have become increasingly popular owing to their ability to use complex datasets and develop predictive algorithms with high precision. Therefore, these models may help predict skin and subcutaneous tissue adverse events in RA patients receiving anti-TNF-α treatment in real clinical settings. Furthermore, a machine learning-based risk score system developed in this study can be potentially applied by clinicians to predict and prevent such complications. This system appears suitable for identifying high-risk patients for close monitoring in the actual clinical setting. These methods should be tested in other settings, appropriate outcome measures should be established to determine the need for rigorous monitoring, and the clinical impact of interventions should be examined.

The limitations of this study encompass its retrospective design and the absence of detailed mechanisms. Additionally, the study exhibited large confidence intervals, possibly stemming from a small sample size. Another limitation of this study is the small number of patients (11 out of 113) with skin and subcutaneous adverse events, preventing a detailed analysis of individual conditions. In addition, the small sample size, especially the limited number of cases, made it impractical to conduct a subgroup analysis to determine if the results vary based on the type of TNF-α inhibitor used. Moreover, considering external validation and enhancing models by integrating more predictors is imperative for machine learning algorithms. Nevertheless, to the best of our knowledge, this study represents the initial exploration of genetic variations’ effects on TNF-α inhibitor-induced skin and subcutaneous tissue-related adverse events in Asian patients. The practical implementation of genome-wide genotyping in clinical settings is crucial for facilitating personalized pharmacotherapy and improving patient outcomes by enabling the timely identification of genetic predispositions to severe adverse skin reactions. Furthermore, it introduces predictive models for cutaneous complications employing machine learning techniques like logistic regression, elastic net, random forest, and SVM. Clinicians can utilize the risk score system developed here to predict adverse events in clinical settings. These findings serve as preliminary evidence, potentially informing tailored intervention strategies to prevent skin and subcutaneous tissue complications in RA patients undergoing anti-TNF-α medications. Moreover, the potential extensibility of our findings to other rheumatic diseases treatable with TNF-α inhibitors, such as ankylosing spondylitis and psoriatic arthritis, is promising. Given the shared inflammatory pathways and treatment modalities, the identified genetic markers may also be relevant for these conditions, though further validation studies are needed.

## 5. Conclusions

In conclusion, the findings emphasize the clinical relevance of understanding genetic predispositions to dermatological complications in RA patients treated with TNF-α inhibitors. This study also highlights the utility of machine learning methods in developing predictive models, with various algorithms showing good performance in risk prediction. These models can potentially be applied in clinical settings to identify high-risk patients, enabling personalized treatment plans and close monitoring to prevent adverse skin events. This also introduces a practical risk prediction tool that clinicians can use to enhance patient care. Future research should focus on elucidating the mechanisms behind these genetic associations and expanding the predictive models to include additional predictors and external validation.

## Figures and Tables

**Table 1:** Factors associated with skin and subcutaneous adverse outcome in rheumatoid arthritis patients receiving TNF-α inhibitors.

		Outcome (n = 11)	No Outcome (n = 102)	*p*-Value
Sex				0.69
	Male	1 (9.1)	21 (20.6)	
	Female	10 (90.9)	81 (79.4)	
Age, years				1.00
	<65	9 (81.8)	84 (82.4)	
	≥65	2 (18.2)	18 (17.6)	
Body Mass Index, m^2^/kg				1.00
	<23	6 (54.5)	58 (56.9)	
	≥23	5 (45.5)	44 (43.1)	
Duration of rheumatoid arthritis, months		117.36 ± 67.686	103.02 ± 78.518	0.81
Rheumatoid factor				0.12
	Positive	11 (100.0)	77 (75.5)	
	Negative	0 (0.0)	25 (24.5)	
ACPA				0.73
	Positive	7 (63.6)	73 (71.6)	
	Negative	4 (36.4)	29 (28.4)	
**Medications**				
Hydroxychloroquine				0.042
	No	2 (18.2)	51 (50.5)	
	Yes	9 (81.8)	50 (49.5)	
Leflunomide				0.20
	No	4 (36.4)	57 (56.4)	
	Yes	7 (63.6)	44 (43.6)	
Methotrexate				0.12
	No	3 (27.3)	10 (9.9)	
	Yes	8 (72.7)	91 (90.1)	
Sulfasalazine				0.08
	No	11 (100)	78 (77.2)	
	Yes	0 (0)	23 (22.8)	
Tacrolimus				0.39
	No	8 (72.7)	85 (84.2)	
	Yes	3 (27.3)	16 (15.8)	
**Comorbidities**				
Gastroduodenitis				0.27
	No	10 (90.9)	99 (98)	
	Yes	1 (9.1)	2 (2)	
Gastroesophageal reflux disease				0.59
	No	11 (100)	91 (90.1)	
	Yes	0 (0)	10 (9.9)	
Headache				1.00
	No	11 (100)	95 (94.1)	
	Yes	0 (0)	6 (5.9)	
Hyperlipidemia				0.63
	No	9 (81.8)	90 (88.2)	
	Yes	2 (18.2)	12 (11.8)	
Hypertension				0.69
	No	9 (81.8)	86 (84.3)	
	Yes	2 (18.2)	16 (15.7)	
Insomnia				0.47
	No	10 (90.9)	96 (95)	
	Yes	1 (9.1)	5 (5)	
Osteoporosis				0.35
	No	11 (100)	88 (86.3)	
	Yes	0 (0)	14 (13.7)	
Rheumatoid lung disease				0.27
	No	10 (90.9)	99 (98)	
	Yes	1 (9.1)	2 (2)	
Tuberculosis				1.00
	No	11 (100)	99 (98)	
	Yes	0 (0)	2 (2)	
Type 2 diabetes mellitus				1.00
	No	11 (100)	96 (94.1)	
	Yes	0 (0)	6 (5.9)	
Vitamin D deficiency				0.26
	No	9 (81.8)	93 (92.1)	
	Yes	2 (18.2)	8 (7.9)	

**Table 2:** Genotype association with TNF-α inhibitor-induced skin and subcutaneous adverse outcome in rheumatoid arthritis patients.

	Genotype	Outcome (n = 11)	No Outcome (n = 102)	*p*-Value
**FRMD3**				
rs12551103				0.001
	CC, CT	7 (63.6)	100 (98)	
	TT	4 (36.4)	2 (2)	
**RP11-1082L8.3**				
rs13265933				0.000166
	GG	2 (18.2)	79 (77.5)	
	GA, AA	9 (81.8)	23 (22.5)	
**RP11-245M24.1**				
rs73210737				0.000419
	TT	4 (36.4)	89 (87.3)	
	CT, CC	7 (63.6)	13 (12.7)	
**33kb 3′ of RP11-425E13.1**				
rs920388				0.003
	GG, GT	8 (72.7)	101 (99)	
	TT	3 (27.3)	1 (1)	

FRMD3: FERM Domain Containing 3.

**Table 3:** Multivariate analysis to identify predictors for skin and subcutaneous adverse outcomes in rheumatoid arthritis patients receiving TNF-α inhibitors.

	Crude OR (95% CI)	Adjusted OR (95% CI)	Score
Sex (female)	2.593 (0.314–21.404)	-	-
Age ≥ 65	1.037 (0.206–5.212)	-	-
Hydroxychloroquine	4.590 (0.944–22.308)	-	-
rs12551103 TT	28.571 (4.437–183.964)	20.137 (1.129–359.238)	4
rs13265933 A	15.457 (3.117–76.633)	14.336 (1.468–140.013)	3
rs73210737 C	11.981 (3.077–46.649)	10.140 (1.502–68.435)	2
rs920388 TT	37.875 (3.523–407.155)	15.916 (0.874–289.792)	3

Adjusted for rs12551103, rs13265933, rs73210737, rs920388, sex, age, and hydroxychloroquine. OR: odds ratio; CI: confidence interval.

## Data Availability

The datasets generated or analyzed during the current study are not publicly available due to their containing information that could compromise the privacy of research participants but are available from the corresponding author upon reasonable request.

## Supplementary Materials

The following supporting information can be downloaded at: https://www.mdpi.com/article/10.3390/jcm13144050/s1.

## References

[1] Malm K., Bergman S., Andersson M.L., Bremander A., Larsson I. (2017). Quality of life in patients with established rheumatoid arthritis: A phenomenographic study. SAGE Open Med..

[2] Symmons D., Mathers C., Pfleger B. (2015). The Global Burden of Rheumatoid Arthritis in the Year 2000 Global Burden of Disease.

[3] Kleinert S., Tony H.P., Krause A., Feuchtenberger M., Wassenberg S., Richter C., Röther E., Spieler W., Gnann H., Wittig B.M. (2012). Impact of patient and disease characteristics on therapeutic success during adalimumab treatment of patients with rheumatoid arthritis: Data from a German noninterventional observational study. Rheumatol. Int..

[4] Haworth C., Brennan F.M., Chantry D., Turner M., Maini R.N., Feldmann M. (1991). Expression of granulocyte-macrophage colony-stimulating factor in rheumatoid arthritis: Regulation by tumor necrosis factor-α. Eur. J. Immunol..

[5] Vasanthi P., Nalini G., Rajasekhar G. (2007). Role of tumor necrosis factor-alpha in rheumatoid arthritis: A review. APLAR J. Rheumatol..

[6] Kerbleski J.F., Gottlieb A.B. (2009). Dermatological complications and safety of anti-TNF treatments. Gut.

[7] Flendrie M., Vissers W.H., Creemers M.C., de Jong E.M., van de Kerkhof P.C., van Riel P.L. (2005). Dermatological conditions during TNF-α-blocking therapy in patients with rheumatoid arthritis: A prospective study. Arthritis Res. Ther..

[8] Lee H.H., Song I.H., Friedrich M., Gauliard A., Detert J., Röwert J., Audring H., Kary S., Burmester G.R., Sterry W. (2007). Cutaneous side-effects in patients with rheumatic diseases during application of tumour necrosis factor-α antagonists. Br. J. Dermatol..

[9] Nigam G.B., Bhandare A.P., Antoniou G.A., Limdi J.K. (2021). Systematic review and meta-analysis of dermatological reactions in patients with inflammatory bowel disease treated with anti-tumour necrosis factor therapy. Eur. J. Gastroenterol. Hepatol..

[10] Pasadyn S.R., Knabel D., Fernandez A.P., Warren C.B. (2020). Cutaneous adverse effects of biologic medications. Cleve Clin. J. Med..

[11] Fréling E., Baumann C., Cuny J.F., Bigard M.A., Schmutz J.L., Barbaud A., Peyrin-Biroulet L. (2015). Cumulative incidence of, risk factors for, and outcome of dermatological complications of anti-TNF therapy in inflammatory bowel disease: A 14-year experience. Am. J. Gastroenterol..

[12] Tillack C., Ehmann L.M., Friedrich M., Laubender R.P., Papay P., Vogelsang H., Stallhofer J., Beigel F., Bedynek A., Wetzke M. (2014). Anti-TNF antibody-induced psoriasiform skin lesions in patients with inflammatory bowel disease are characterised by interferon-γ-expressing Th1 cells and IL-17A/IL-22-expressing Th17 cells and respond to anti-IL-12/IL-23 antibody treatment. Gut.

[13] Rahier J.F., Buche S., Peyrin-Biroulet L., Bouhnik Y., Duclos B., Louis E., Papay P., Allez M., Cosnes J., Cortot A. (2010). Severe skin lesions cause patients with inflammatory bowel disease to discontinue anti-tumor necrosis factor therapy. Clin. Gastroenterol. Hepatol..

[14] Broge T., Nguyen N., Sacks A., Davis M. (2013). Infliximab-associated psoriasis in children with Crohn’s disease may require withdrawal of anti-tumor necrosis factor therapy. Inflamm. Bowel Dis..

[15] National Cancer Institute (2017). Common Terminology Criteria for Adverse Events (CTCAE) v5.0.

[16] Moustou A.E., Matekovits A., Dessinioti C., Antoniou C., Sfikakis P.P., Stratigos A.J. (2009). Cutaneous side effects of anti-tumor necrosis factor biologic therapy: A clinical review. J. Am. Acad. Dermatol..

[17] Andrade P., Lopes S., Gaspar R., Nunes A., Magina S., Macedo G. (2018). Anti-Tumor Necrosis Factor-α-Induced Dermatological Complications in a Large Cohort of Inflammatory Bowel Disease Patients. Dig. Dis. Sci..

[18] Seneschal J., Milpied B., Vergier B., Lepreux S., Schaeverbeke T., Taïeb A. (2009). Cytokine imbalance with increased production of interferon-alpha in psoriasiform eruptions associated with antitumour necrosis factor-alpha treatments. Br. J. Dermatol..

[19] Collamer A.N., Battafarano D.F. (2010). Psoriatic skin lesions induced by tumor necrosis factor antagonist therapy: Clinical features and possible immunopathogenesis. Semin. Arthritis Rheum..

[20] de Gannes G.C., Ghoreishi M., Pope J., Russell A., Bell D., Adams S., Shojania K., Martinka M., Dutz J.P. (2007). Psoriasis and pustular dermatitis triggered by TNF-{alpha} inhibitors in patients with rheumatologic conditions. Arch. Dermatol..

[21] Wollina U., Hansel G., Koch A., Schönlebe J., Köstler E., Haroske G. (2008). Tumor necrosis factor-alpha inhibitor-induced psoriasis or psoriasiform exanthemata: First 120 cases from the literature including a series of six new patients. Am. J. Clin. Dermatol..

[22] Fidder H., Schnitzler F., Ferrante M., Noman M., Katsanos K., Segaert S., Henckaerts L., Van Assche G., Vermeire S., Rutgeerts P. (2009). Long-term safety of infliximab for the treatment of inflammatory bowel disease: A single-centre cohort study. Gut.

[23] Askling J., Fahrbach K., Nordstrom B., Ross S., Schmid C.H., Symmons D. (2011). Cancer risk with tumor necrosis factor alpha (TNF) inhibitors: Meta-analysis of randomized controlled trials of adalimumab, etanercept, and infliximab using patient level data. Pharmacoepidemiol. Drug Saf..

[24] Pezzolesi M.G., Poznik G.D., Mychaleckyj J.C., Paterson A.D., Barati M.T., Klein J.B., Ng D.P., Placha G., Canani L.H., Bochenski J. (2009). Genome-wide association scan for diabetic nephropathy susceptibility genes in type 1 diabetes. Diabetes.

[25] Bin L., Malley C., Taylor P., Preethi Boorgula M., Chavan S., Daya M., Mathias M., Shankar G., Rafaels N., Vergara C. (2021). Whole genome sequencing identifies novel genetic mutations in patients with eczema herpeticum. Allergy.

[26] Hellwege J.N., Russell S.B., Williams S.M., Edwards T.L., Velez Edwards D.R. (2018). Gene-based evaluation of low-frequency variation and genetically-predicted gene expression impacting risk of keloid formation. Ann. Hum. Genet..

