# Supplementary material for: Development of a Risk Prediction Model for Adverse Skin Events Associated with TNF-α Inhibitors in Rheumatoid Arthritis Patients
Source: J Clin Med. 2024 Jul 11;13(14):4050. doi: 10.3390/jcm13144050 (PMC11278277; doi:10.3390/jcm13144050)
Supplement: Supplementary file 1 [file jcm-13-04050-s001.zip › jcm-3092766-supplementary.pdf]

Supplementary Materials

Table S1. Baseline DAS28 with its subcomponents.

	Outcome (n=11)	No outcome (n=102)	p-value
**Baseline DAS28 with its subcomponents**			
DAS28	5.67±1.286	5.76±1.042	0.541
Tender joint count 28	12.18±10.980	10.00±6.834	0.087
Swollen joint count 28	8.55±10.530	7.04±5.179	0.070
Global health	56.36±16.293	59.45±20.312	0.356
ESR	44.82±35.510	49.29±27.394	0.432
CRP	3.32±6.759	2.42±2.923	0.011

DAS: disease activity score; ESR: erythrocyte sedimentation rate; CRP: c-reactive protein; ACPA: anti-citrullinated protein/peptide antibody.

Table S2. Risk of skin and subcutaneous adverse outcome according to scores using logistic regression.

Score	0	2	3	4	5	6	8	9	10
Risk Probability (%)	0.6	3.6	8.5	18.9	36.7	59.2	90.0	95.7	98.2

Table S3. Comparisons of AUC for logistic regression, elastic net, random forest, and SVM models

	AUROC	95% CI	AUPRC	95% CI
**GLM**	0.90	0.768–1.030	0.80	0.630–0.976
**Elastic net**	0.90	0.777–1.030	0.84	0.679–0.992
**Random forests**	0.88	0.740–1.010	0.80	0.633–0.970
**SVM with linear kernel**	0.89	0.760–1.020	0.79	0.620–0.970
**SVM with radial kernel**	0.90	0.768–1.030	0.80	0.636–0.969

AUROC: area under the receiver-operating curve; CI: confidence interval; GLM: generalized linear model SVM: support vector machine

Table S4. Machine learning model specifics

Method	Hyperparameter<br>Model Specification and Search Grids	Selected Values
Elastic net	λ: 100 equally spaced values in logarithmic scale between 10^{-4} and 0	λ: 0.1707353
	α: 0, 0.2, 0.4, 0.6, 0.8, 1	α: 0
Random forests	mtry: 1, 2, 3, 4	mtry: 1
SVM with linear kernel	C: 0, 0.001, 0.005, 0.01, 0.05, 0.1, 0.25, 0.5, 0.75, 1, 1.25, 1.5, 1.75, 2, 5	C: 0.001
SVM with radial kernel	Sigma: 2^{-15}, 2^{-13}, 2^{-11}, 2^{-9}, 2^{-7}, 2^{-5}, 2^{-3}, 2^{-1}, 2, 2^{3}	Sigma: 0.001953125
	C: 2^{-5}, 2^{-3}, 2^{-1}, 2, 2^{3}, 2^{5}, 2^{7}, 2^{9}, 2^{11}, 2^{13}, 2^{15}	C: 8

SVM: Support vector machine
